# Immune-Checkpoint Inhibitors in B-Cell Lymphoma

**DOI:** 10.3390/cancers13020214

**Published:** 2021-01-08

**Authors:** Marc Armengol, Juliana Carvalho Santos, Miranda Fernández-Serrano, Núria Profitós-Pelejà, Marcelo Lima Ribeiro, Gaël Roué

**Affiliations:** 1 Lymphoma Translational Group, Josep Carreras Leukaemia Research Institute (IJC), 08916 Badalona, Spain; marmengol@carrerasresearch.org (M.A.); mfernandez@carrerasresearch.org (M.F.-S.); nprofitos@carrerasresearch.org (N.P.-P.); mlima@carrerasresearch.org (M.L.R.); 2 Laboratory of Immunopharmacology and Molecular Biology, Sao Francisco University Medical School, Braganca Paulista, São Paulo 01246-100, Brazil

**Keywords:** immune checkpoint, lymphoid neoplasms, programmed death 1, cytotoxic T-lymphocyte antigen 4, monoclonal antibodies, combination therapies

## Abstract

**Simple Summary:**

Immune-based treatment strategies, which include immune checkpoint inhibition, have recently become a new frontier for the treatment of B-cell-derived lymphoma. Whereas checkpoint inhibition has given oncologists and patients hope in specific lymphoma subtypes like Hodgkin lymphoma, other entities do not benefit from such promising agents. Understanding the factors that determine the efficacy and safety of checkpoint inhibition in different lymphoma subtypes can lead to improved therapeutic strategies, including combinations with various chemotherapies, biologics and/or different immunologic agents with manageable safety profiles.

**Abstract:**

For years, immunotherapy has been considered a viable and attractive treatment option for patients with cancer. Among the immunotherapy arsenal, the targeting of intratumoral immune cells by immune-checkpoint inhibitory agents has recently revolutionised the treatment of several subtypes of tumours. These approaches, aimed at restoring an effective antitumour immunity, rapidly reached the market thanks to the simultaneous identification of inhibitory signals that dampen an effective antitumor response in a large variety of neoplastic cells and the clinical development of monoclonal antibodies targeting checkpoint receptors. Leading therapies in solid tumours are mainly focused on the cytotoxic T-lymphocyte-associated antigen 4 (CTLA-4) and programmed death 1 (PD-1) pathways. These approaches have found a promising testing ground in both Hodgkin lymphoma and non-Hodgkin lymphoma, mainly because, in these diseases, the malignant cells interact with the immune system and commonly provide signals that regulate immune function. Although several trials have already demonstrated evidence of therapeutic activity with some checkpoint inhibitors in lymphoma, many of the immunologic lessons learned from solid tumours may not directly translate to lymphoid malignancies. In this sense, the mechanisms of effective antitumor responses are different between the different lymphoma subtypes, while the reasons for this substantial difference remain partially unknown. This review will discuss the current advances of immune-checkpoint blockade therapies in B-cell lymphoma and build a projection of how the field may evolve in the near future. In particular, we will analyse the current strategies being evaluated both preclinically and clinically, with the aim of fostering the use of immune-checkpoint inhibitors in lymphoma, including combination approaches with chemotherapeutics, biological agents and/or different immunologic therapies.

## 1. Biology of B-Cell Lymphoma

The term B-cell lymphoma encompasses different neoplasms characterised by an abnormal proliferation of lymphoid cells at various stages of differentiation. B-cell lymphoma develops more frequently in older adults and immunocompromised individuals and includes both Hodgkin’s lymphomas (HLs) and most B-cell non-Hodgkin lymphomas (B-NHLs). The latter accounts for up to 4% of the globally diagnosed cancers [1] and are characterised by a malignant proliferation of mature or immature B-lymphocytes in lymphoid tissues and extranodal territories such as the gastrointestinal tract, the central nervous system (CNS), or, essentially, any other body organ [2]. Inherited events such as chromosomal translocations, oncogene activation or even certain viral infections such as the Epstein–Barr virus (EBV) may trigger lymphomagenesis [3]. B-NHLs are divided into low and high grades, typically corresponding to indolent (slow-growing) lymphomas and aggressive lymphomas, respectively. Indolent lymphomas include follicular lymphoma (FL), marginal zone lymphoma (MZL), small cell lymphocytic lymphoma (SLL)/chronic lymphocytic leukaemia (CLL) and Waldenström macroglobulinemia (WM). Early-stage indolent B-cell lymphomas can often be treated with radiation alone, with long-term nonrecurrence. Early-stage aggressive disease is treated with chemotherapy and, often, radiation, with a 70–90% curation rate. Aggressive lymphomas include both precursor lymphoid neoplasms and numerous mature B-cell neoplasms like mantle cell lymphoma (MCL), primary effusion lymphoma (PEL), Burkitt lymphoma (BL), diffuse large B-cell lymphoma (DLBCL) and its many subtypes and variants, and unclassifiable B-cell lymphoma, with features intermediate between DLBCL and BL. These entities usually require intensive treatments, with some having a good prospect for a permanent cure [4].

### 1.1. Diffuse Large B-Cell Lymphoma

Diffuse large B-cell lymphoma (DLBCL) represents the most common type of B-NHL in Western countries. The 2016 World Health Organization (WHO) classification of lymphoid malignancies recognises several subtypes characterised by unique clinical and pathological features, including primary DLBCL of the central nervous system (PCNSL), primary cutaneous DLBCL, leg type, T-cell/histiocyte-rich large cell lymphoma, and EBV-positive DLBCL of the elderly. Nevertheless, most cases of DLBCL fall into the “not otherwise specified” (NOS) category [4].

DLBCL, like other cancers, develops in a complex tissue environment with a high content of malignant and nonmalignant compartments of the disease, as well as extracellular components that constitute the tumour microenvironment (TME). The cellular and molecular features of TME have a profound prognostic impact [5] and include T-cells, tumour-associated macrophages (TAMs), dendritic cells (DCs), neutrophils, natural killer (NK) cells and stromal cells [6]. DLBCL harbours a noninflamed phenotype characterised by lack of immune cell infiltration, which could explain the modest efficacy of immune checkpoint blockade therapy in relapsed/refractory (R/R) DLBCL patients [7]. 

### 1.2. Primary Mediastinal B-Cell Lymphoma

Primary mediastinal B-cell lymphoma (PMBL) is a rare but aggressive lymphoma of thymic B-cell origin, accounting for 3% of B-NHLs. Although it presents similar histology to DLBCL, the genetic profile of PMBL is distinct and shares many features with classic Hodgkin lymphoma (cHL, see below) [8]. Patients are generally not cured after first-line treatment, and, after relapse, autologous stem cell transplantation (ASCT) is usually beneficial. However, relapsed/refractory (R/R) PMBL cases have poor outcomes and are often managed like other forms of DLBCL [9].

### 1.3. Follicular Lymphoma

Follicular lymphoma (FL) is the second most common B-NHL, accounting for 29–35% of cases. It is a neoplasm of germinal centre B-cells, which display rearrangement of immunoglobulin (Ig) heavy and light chain genes and somatic hypermutation and express common germinal centre markers such as BCL6, AID and CD10 [10,11,12]. FL generally presents an indolent clinical course, with median overall survival (OS) of more than 15 years [10,13]. However, about 20% of patients relapse during the first 2 years after treatment, and others evolve into transformed-FL (t-FL), a much more aggressive subtype [10].

The crosstalk between malignant FL cells and the surrounding cells of their TME is driven by some recurrent genetic events [14]. FL is strongly regulated by direct interaction with a germinal centre (GC)-like microenvironment, including myeloid cells, follicular helper T-cells (T_FH_), and stromal cells, that may orchestrate efficient immune escape mechanisms [15]. The TME of FL also displays deregulation of the extracellular matrix proteins involved in collagen deposition and organization [16]. Cancer-associated fibroblasts (CAFs) are another important FL tumour-promoting actor, providing a niche with high levels of factors involved in B-cell activation and the activation/recruitment of some TME components such as TAMs [17]. The crosstalk between T_FH_ cells and FL cells is orchestrated by the interaction between antigen-loaded MHC class II molecules and antigen-specific T-cell receptors. 

### 1.4. Burkitt Lymphoma

Burkitt lymphoma (BL) includes a heterogeneous group of highly aggressive malignancies of intermediate-sized B-cells that may be found infiltrating both nodal or extranodal tissues in a diffuse pattern [18]. BL is invariably associated with chromosomal translocations that dysregulate the expression of c-MYC, and, consequently, several downstream genes involved in the control of cellular processes such as cell cycle progression and apoptosis [19]. The malignant cells usually express the B-cell-specific surface markers CD19 and CD20, as well as low-to-intermediate levels of common acute lymphoblastic leukaemia (ALL) antigen (CD10/CALLA) [20].

The complex interplay between BL cells and the TME also regulates lymphomagenesis and provides new insights for target immunotherapies. Like DLBCL, BL tumours harbour a noninflamed environment with low infiltration of immune cells and are usually resistant to immune checkpoint blockade. One of the hallmarks of the TME in BL tumours is the high content of TAMs which contribute to tumour progression through the secretion of cytokines and chemokines, and the expression of immune checkpoint proteins such as programmed death ligand 1 (PD-L1) [21] (see below). The crosstalk between tumour cells, TAMs, PD-1 signalling, viral antigens, and T-cells may result in the high prevalence of M2 macrophages in the TME and contribute to the failed immunity of BL patients [22].

### 1.5. Marginal Zone Lymphoma

Marginal zone lymphoma (MZL) originates from memory B-cells at the marginal zone of lymphoid follicles and account for 5–15% of all NHLs [23,24]. Three distinct entities have been described. Splenic (SMZL) and nodal marginal zone lymphoma (NMZL) arise from the follicle marginal zone of the spleen and the lymph nodes, respectively [24,25,26]. Extranodal marginal zone lymphoma (EMZL) of the mucosa-associated lymphoid tissue (MALT) is the most common subtype, accounting for about 60% of MZL cases. This entity is strongly associated with chronic inflammation derived from autoimmune disease or infection, such as *Helicobacter pylori*. Other tumours sites include eyes and ocular adnexa (13%), skin (9%), lungs (9%) and salivary glands (8%) [23,27,28]. MZLs mostly have indolent clinical courses, although NMZL has a poorer prognosis than other subtypes [25,26,27].

The course of MZL disease is strongly influenced by the TME, and this latter may therefore represent a promising strategy for early diagnosis and therapy choice. SMZL cells are supported by immune cells such as mast cells and macrophages, which may be recruited by tumour cells through the secretion of cytokines and chemokines [29]. The TME components of SMZL can regulate stromal cell proliferation, angiogenesis, extracellular matrix remodelling, and induction of adhesion molecule expression [29]. The chronic inflammation of MALT lymphomas not only triggers B-cell growth but also recruits T-cells, macrophages and neutrophils to the site of inflammation, which contribute to genetic aberrations, DNA damage and genetic instability of the B-cells during somatic hypermutation and class-switching recombination [30]. 

### 1.6. Mantle Cell Lymphoma

Mantle cell lymphoma (MCL) originates from B-cells, a proportion of them being antigen-experienced B-cells, in the mantle zone of lymph nodes. MCL is usually diagnosed as a late-stage disease and may be observed in both the gastrointestinal tract and bone marrow [31]. The diagnosis of MCL is mainly performed by a microscopic evaluation of a biopsy, although the detection of chromosomal translocation t (11:14), with the consequent cyclin D1 expression, is considered the molecular hallmark [32].

The crosstalk between MCL tumour cells and its microenvironment has a central role in disease expansion [33]. MCL cells have shown constitutive expression of PD-1 and its ligand PD-L1, which converts it into an interesting candidate for immunotherapy targeting this checkpoint [34] (see below). Aggressive MCL cases are characterised by a low number of T-cells [35] and a high frequency of regulatory T-cells (Treg) [36]. Moreover, follicular dendritic cells (FDCs) have been shown to support MCL cell survival through a cell–cell interaction mechanism [37]. Autocrine and paracrine secretion of soluble factors could also have an important role within the MCL TME. Interestingly, the blood of MCL patients contains high levels of several cytokines and chemokines, such as IL-8, CCL3 and CCL4, which are correlated with poor survival [38]. 

### 1.7. Classical Hodgkin Lymphoma

Classical Hodgkin lymphoma (cHL) is a neoplasm derived from B-cells and is mainly constituted by a small number of neoplastic mononuclear cells, i.e., Hodgkin cells, and multinucleated Reed–Sternberg (HRS) cells. cHL accounts for 15–25% of all lymphomas and represents the most common lymphoma subtype in children and young adults in the Western world. The cell of origin (COO) is nowadays unequivocally considered to be a (post)germinal centre B-cell [39]. Several genetic alterations, targeting a few pathways, have been identified, but none of them can be considered “dominant”. The affected pathways include NF-κB and JAK-STAT, whose aberrant activation fuel HRS cells with proliferative and antiapoptotic stimuli [40]. Moreover, the LMP1 protein, encoded by EBV that often latently infects HRS cells, likely contributes to NF-κB signalling since LMP1 mimics constitutively active CD402 [41].

Genetic lesions of NF-κB pathway genes largely contribute to aberrant activation of this cascade in a cell-intrinsic manner and/or by amplifying signals from the microenvironment [42]. In addition, HRS cells are outnumbered by reactive cells in the TME, including T- and B-lymphocytes, eosinophils, macrophages, mast cells, plasma cells and stromal cells [43,44].

## 2. Immune Checkpoint Blockade in B-Cell Lymphoma

**Figure 1:**
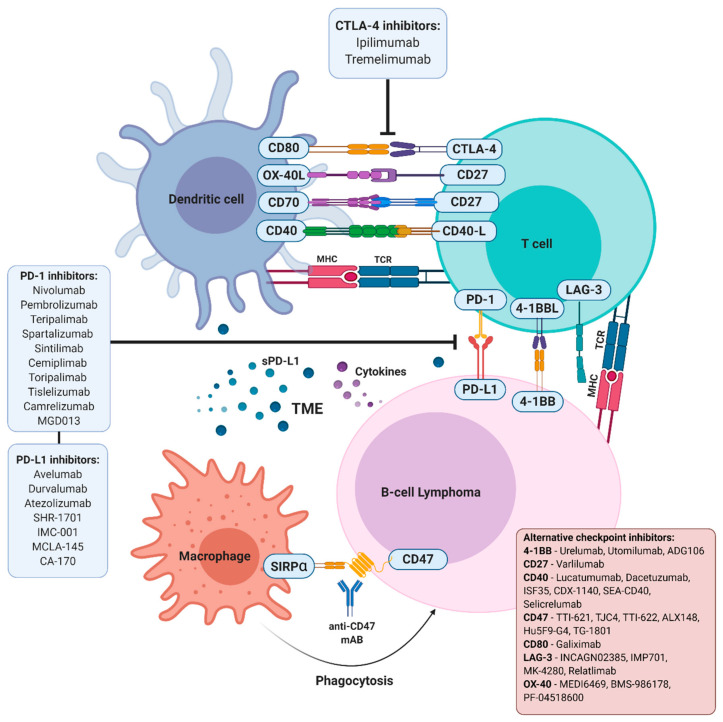
Therapeutic approaches based on immune checkpoint blockade in B-cell lymphomas. Different therapeutic strategies to block PD-1/PD-L1 interaction are under clinical development in order to prevent PD-1-mediated attenuation of TCR signalling, allowing for activity restoration of exhausted CD8+ T-cells. CTLA-4 inhibition by monoclonal antibodies may induce tumour rejection through direct blockade of CTLA-4 competition for CD-80 (B7-1) and CD-86 (B7-2) ligands, which enhances CD28 costimulation and, thus, activation. Alternative immune checkpoint molecules expressed on tumour cells or immune cells in the TME can be simultaneously modulated to restore an effective antilymphoma immune response.

Among the armament of immunotherapies aimed at allowing the host’s own immune system to detect and eliminate malignant cells, immune checkpoints blockers are able to modulate molecules that regulate immune signalling, either positively, by promoting the activation, maturation and proliferation of T-cells, or negatively, by blocking T-cell activity, eventually leading to the programmed death of these latter (Figure 1). Most B-NHLs, including BL, DLBCL, FL and CLL, are characterised by a low infiltration of immune cells, a feature that may condition a priori the applicability of immune checkpoint blockers. Although there is no evidence of a specific genetic immune escape program that may prevent immune cells from entering the local TME to promote an effective antitumor response in a determined lymphoma subtype, recent data support the notion that oncogenic signalling can promote a “noninflamed” TME. As an example, *PTEN*, *EZH2*, and *TP53* dysregulation have been associated with the downregulation of genes related to innate or adaptive immunity in DLBCL, potentially leading to immune suppression, decreased HLA expression and reduced T-cell infiltration [45,46,47,48,49,50]. The oncogene *MYC*, involved in the pathogenesis of BL and other lymphoma subtypes, may also be involved in the regulation of the immune environment by regulating the transcription of different immune checkpoint molecules, including CD47 and PD-L1 [51].

### 2.1. PD-1/PD-L1 Blockade

#### 2.1.1. PD-1 Signalling Overview

Overexpression of PD-1 and its ligands, PD-L1 (CD274) and/or PD-L2 (PDCD1LG2), by malignant neoplastic cells allows the ligation of PD-1 on T-cells and the consequent induction of T-cell “exhaustion”, a phenomenon closely linked to peripheral tolerance and homeostasis. That way, the malignant cells escape from the antitumor immune response in a process known as immune evasion [52].

PD-1 is a protein encoded by the *PDCD1* gene at chromosome 2q37.3, which contains an extracellular domain, a transmembrane domain, and a cytoplasmic domain with two tyrosine signalling motifs [53]. PD-1 is expressed on CD4+ and CD8+ T-cells, B-cells, NK cells, macrophages, and some DCs during immune activation and inflammation [54,55]. On B-cells, PD-1 is markedly regulated by B-cell receptor (BCR) signalling, lipopolysaccharide (LPS), CpG oligodeoxynucleotides, and several proinflammatory cytokines [56] (Figure 1). 

The PD-L1 protein is encoded by the *CD274* gene on chromosome 9p24.1 and harbours two extracellular domains, a transmembrane domain, and a short cytoplasmic tail that lacks signalling motifs [57]. The expression of PD-L1 is strongly affected by structural alterations such as amplifications, gains, and translocations of chromosome 9p24.1 [58]. Remarkably, 9p24.1 amplification also induces Janus kinase 2 (JAK2) expression, leading to activation of JAK/signal transducers and activators of transcription (STAT) signalling, which in turn, upregulates PD-L1 [41]. Upon engagement with PD-L1, PD-1 becomes phosphorylated by Src family kinases and transmits a negative costimulatory signal through tyrosine phosphatase proteins to attenuate the strength of T-cell receptor (TCR) signals and downstream signalling pathways such as PTEN–PI3K–AKT and RAS–MEK–ERK. The functional outcome of this regulation is the inhibition of cytotoxic T-lymphocyte function [59,60,61,62,63].

In 70–87% of cHL patients, PD-L1 is detected on the surface of both HRS cells and TAMs [64,65,66,67,68] and is associated with worse event-free survival (EFS) and shorter progression-free survival (PFS) [64]. This overexpression can be consequent to EBV infection [69]; in a large majority of cases, PDL-1 upregulation is the result of genetic alterations of chromosome 9p24.1, thereby also affecting the expression of PDL-2 and JAK2 [41,64,66,68]. Increased PDL-1 expression by TAMs following interferon (IFN)-γ signalling may be particularly relevant in cHL clinical outcomes due to the close relationship between HRS and PD-1+ CD4+ T-cells [70,71].

In DLBCL, PD-L1 has been shown to be expressed by the nonmalignant compartment in only 26% to 75% of the cases [65,72,73,74,75]. Godfrey et al. showed that 27% of DLBCL patients (especially from the nongerminal centre subgroup) presented a PD-L1 amplification associated with inferior PFS following front-line chemoimmunotherapy [58,71,72,74,76,77,78]; this was more often detected in de-novo than transformed cases [65,76]. Similar to cHL, EBV infection has been correlated with a much higher PD-L1 expression in DLBCL tumours [74]. The prognostic significance of PD-L1 expression in DLBCL patients is controversial, but most of the studies have reported a poorer outcome in cases with PD-L1+ macrophages [74]. Additionally, overexpression of PD-L1 is associated with the immune escape gene signature involving Bruton’s tyrosine kinase (BTK) and JAK/STAT signalling [79].

Genetic alterations of chromosome 9p24.1 of PD-L1 and/or PD-L2 have also been reported in PMBL, and in two other lymphoma subtypes that arise in immune-privileged extranodal sites, i.e., PCNSL, and primary testicular lymphoma (PTL) [58,71,80,81,82,83]. Accordingly, PD-L1 and PD-L2 are found to be overexpressed in a majority of PMBL patients [41,66,71,84] and about 50% of PCNSL and PTL patients harbour PD-1 ligand overexpression [80].

Regarding PD-1, receptor expression was detected in 39.5–68.6% of DLBCL cases [85], and data support the notion that a high number of PD-1+ tumour-infiltrating lymphocytes (TILs) are associated with favourable clinical features and prognosis [72,86]. In contrast to DLBCL, FL tumour cells are largely negative for PD-L1 and PD-L2, and in this disease, the TILS are characterised by high PD-1 expression and suppressed cytokine signalling [87]. Importantly, the presence of PD-1+ TILs is a favourable prognostic factor, whereas a low number of TILs is associated with increased risk of histologic transformation [88,89].

Finally, in MCL, available data on the expression of PD-L1 are often conflicting. Several studies have shown that PD-L1 expression is low or absent in MCL [65,90], whereas others have shown a variable but constitutive expression of PD-L1 on tumour cells in both cell lines and primary patient samples [34].

#### 2.1.2. PD-1/PD-L1 Inhibition in B-Cell Lymphoma

**Figure 2:**
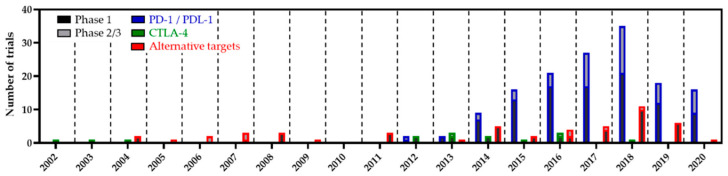
Clinical evolution of immune checkpoint-based therapies in B-cell lymphoma over the last 20 years (according to https://beacon-intelligence.com/checkpoint; data actualised in September 2020).

The blockade of the PD-1/PD-L1 pathway (Figure 2) has transformed immunotherapy with a promising increase in OS rates, leading to U.S. Food and Drug Administration (FDA) approval of these immune checkpoint blockade drugs for the treatment of a broad range of tumour types over the past decade. Two anti-PD-1 antibodies (nivolumab (BMS-936558/ONO-4538, Opdivo^®^) and pembrolizumab (Keytruda^®^)) and three anti-PD-L1 antibodies (durvalumab, atezolizumab, and avelumab) have been approved for the treatment of various types of cancer, including lymphomas [68,91,92,93,94,95,96].

Nivolumab and pembrolizumab, two fully humanised IgG4-kappa-blocking monoclonal antibodies, target the PD-1 receptor on human T-cells [97,98,99]. Nivolumab binds specifically to PD-1 and does not affect the related members of the CD28 family, such as CD28, CTLA-4, inducible co-stimulator, and B- or T-lymphocyte attenuator. The blockade of the PD-1 signalling pathway by nivolumab induces both the proliferation of lymphocytes and the release of IFN-γ. Pembrolizumab binds with high affinity to human PD-1, blocking receptor ligation by both PD-L1 and PD-L2 and leading to enhanced T-lymphocyte immune responses in preclinical models of cancer, with the modulation of key cytokines like interleukin (IL)-2, tumour necrosis factor (TNF)-α, and IFN-γ [100,101].

Most DLBCL patients were initially thought to be not amenable to PD-1 blockade since PDL-1/2 alterations are nonfrequent in this disease, and, accordingly, PD-1 blockade therapy has been disappointing to date in R/R DLBCL and FL. While several ongoing clinical trials are evaluating the use of pembrolizumab in different DLBCL subtypes, this antibody failed to improve PFS in ASCT-relapsed patients [102]. Similarly, a first phase-1b dose-escalation cohort expansion study evaluating nivolumab in R/R DLBCL patients (NCT01592370) and a subsequent larger phase-2 study (NCT02038933) in ASCT-relapsed and ASCT-ineligible DLBCL patients reported overall response rates (ORRs) <40% [78,97,100,103] (Table 1). In contrast, in CLL patients with Richter’s transformation (RT), the recent phase-2 trial, MC1485 (NCT02332980), demonstrated an ORR of 44%, including 1 complete response (CR), 2 partial responses (PR) and median PFS and OS of 5.4 months and 10.7 months, with manageable adverse events (AEs; Table 2). As expected, those patients displayed higher levels of PD-L1 expression related to the presence of chromosome 9p24.1 amplification or EBV infection [104].

Considering the recurrent alteration of the PD-L1 gene in PCNSL and PTL and the poor prognosis of these rare subtypes of DLBCL [80], nivolumab was evaluated in patients with R/R PCNSL or R/R PTL, in whom it demonstrated impressive activity (NCT02857426), with clinical and radiographic response and PFS extended to 13+ to 17+ months for some patients [105]. The phase-2 CheckMate 436 clinical trial further demonstrated that nivolumab combined with brentuximab vedotin represents a promising therapy in PBML patients post-ASCT or after ≥2 prior chemotherapy regimens [106]. Similarly, pembrolizumab therapy has also yielded excellent results in PMBL patients. In the phase-1b multicohort KEYNOTE-013 study, the ORR was 41%, while the median duration of response (DOR) and OS were not reached in this subset of patients [107]. The subsequent international phase-2 KEYNOTE-170 study (NCT02576990), with 53 R/R PBML patients enrolled, reported an ORR of 45% (including 13% CR) [108]. As expected, the magnitude of chromosome 9p24.1 abnormality was associated with PD-L1 expression in responding patients [108]. Importantly, in both KEYNOTE studies, no patient who previously achieved CR relapsed during the follow-up. Altogether, these results led to the accelerated FDA approval of pembrolizumab in 2018 for the treatment of R/R PMBL [99].

Similarly, the phase-1/2 studies employing nivolumab and pembrolizumab have reported high ORRs in patients with R/R cHL; the patients that reached CR were characterised by higher PFS [67,68,109,110,111,112] (Table 1). In subsequent phase-2 studies, pembrolizumab led to 100% OS and 82% PFS at 18 months in post-ASCT consolidation settings, suggesting that pembrolizumab could be used in high-risk patients after ASCT to remodel the immune landscape [113]. Following these trials, pembrolizumab is currently being used in frontline and salvage regimens in R/R cHL patients [114]. 

Two ongoing and three completed clinical trials evaluated the safety and efficacy of the humanised IgG1 monoclonal anti-PD-L1 antibody atezolizumab (MPDL-3280A), in combination with the anti-CD20 antibody obinutuzumab, for the treatment of aggressive B-cell lymphoma. A phase-1/2 trial (NCT02596971) evaluated the safety and efficacy of atezolizumab, in combination with either obinutuzumab + the alkylating agent bendamustine or obinutuzumab + chemotherapy (CHOP) in FL patients, and atezolizumab + rituximab + chemotherapy in DLBCL patients. The analysis from 40 patients demonstrated high efficacy (ORR of 95%) and durable responses (24 months for 80% of patients) for the combinational approach. Another phase-1/2 study (NCT02631577) that enrolled 38 patients with R/R FL demonstrated durable clinical responses and the remarkable ORR of atezolizumab, in combination with obinutuzumab, plus the immunomodulatory drug lenalidomide. Nevertheless, a phase-1 trial (NCT02220842), including 14 patients with R/R FL and 17 patients with R/R DLBCL, showed the weak efficacy of atezolizumab in combination with obinutuzumab or the EZH2 inhibitor tazemetostat. Subsequently, a multicentre phase-2 trial (NCT03276468) assessed the antilymphoma activity of atorolimumab associated with Veneto lax (a BCL-2 inhibitor) and blinatumomab in three cohorts: R/R FL patients, R/R DLBCL patients and iNHLs, including MZL and MALT cases. The data from the 58 DLBCL patients enrolled at the time of the primary analysis demonstrated that the efficacy of combinatory therapy is comparable with currently available options for this population, with durable responses. The phase-1/2 clinical trial NCT02729896 evaluated the combination of atezolizumab with obinutuzumab and polatuzumab, an anti-CD79b, in 13 participants with R/R FL and atezolizumab with the anti-CD20 antibody rituximab and polatuzumab in 21 participants with R/R DLBCL. The percentage of participants with an objective response (CR + PR) was 33.33–57.14% (depending on the polatuzumab dose) for FL patients and 25% for DLBCL patients. The results of a large phase-1 clinical trial (NCT02500407) that enrolled 72 iNHL patients (including 69 FL) and 141 cases with aggressive B-NHL (87 DLBCL and 29 tFL) to evaluate the combination of atezolizumab with mosunetuzumab, a bispecific CD20-CD3 monoclonal antibody, demonstrated high response rates and durable complete remissions, as well as the maximum tolerated dose. The ORR and CR of the iNHL patients across all dose levels were 64% and 42%, respectively. The ORR and CR of aggressive NHL patients across all dose levels were 34.7% and 18.6%, respectively (Table 1).

According to the outcome of the NP39488 study (NCT03533283), the combination of atezolizumab with glofitamab, another bispecific antibody designed to target CD20 on the surface of B-cells and CD3 on the surface of T-cells, resulted in low ORR in 38 aggressive B-NHL patients or iNHL.

The use of CAR-modified T-cells targeting specific tumour cell antigens to enhance immune responses against tumour cells is certainly a great breakthrough in oncoimmunotherapy research. In NHLs, targeting CD19-malignant B-cells has proven highly efficacious in the refractory-disease setting, resulting in T-cell activation, proliferation and secretion of inflammatory cytokines and chemokines, with consequent tumour cell lysis [115,116]. KTE-C19 (Axi-cel) is an autologous anti-CD19 CAR T-cell that was approved by the FDA in October 2017 for the treatment of R/R aggressive B-cell lymphomas after two or more lines of systemic therapy. As PD-1/PD-L1 blockade has been shown to be upregulated after CAR T-cell infusion, the ZUMA-6 clinical trial (NCT02926833) evaluated outcomes of KTE-C19 combined with the anti-PD-L1 atezolizumab. The data suggested that PD-L1 blockade with atezolizumab after KTE-C19 has a manageable safety profile and a promising efficacy outcome (Table 1).

Durvalumab is a selective, high-affinity, humanised IgG1-kappa monoclonal antibody against PD-L1 [117]. In vitro and in vivo xenograft assays have demonstrated that durvalumab evokes a 75% tumour growth reduction in the presence of tumour-reactive human T-cells, supporting the immunological mechanism of action of this drug [118]. Currently, ten clinical trials are underway to investigate the use of durvalumab as monotherapy or in combination with other reagents or CAR T-cells to treat B-NHL patients. Data from murine lymphoma models suggest that the BTK inhibitor ibrutinib, combined with an anti–PD-L1 therapy, may have synergistic antitumor activity [119]. A phase-1b/2 study (NCT02401048) evaluating the efficacy and safety of the combination of ibrutinib and durvalumab in patients with R/R DLBCL or FL has highlighted longer PFS and OS in patients with FL compared to those with DLBCL. However, the efficacy of ibrutinib + durvalumab treatment demonstrated similar activity to single-agent ibrutinib [120] (Table 1). FUSION NHL 001 (NCT02733042) is a phase-1/2, open-label, multicentre study to assess the safety and tolerability of durvalumab as monotherapy or in combination with different regimens (lenalidomide ± rituximab; ibrutinib; rituximab ± bendamustine (an alkylating agent)) in subjects with B-NHL or CLL. From the 106 enrolled participants, 23 were FL, 37 were DLBCL, 17 were MCL, 5 were MZL, 1 was t-FL, 5 were cHL and 18 were CLL/SLL. The efficacy of the durvalumab and rituximab or durvalumab and lenalidomide + rituximab combination was evaluated initially in 3 B-NHL patients. The ORR of durvalumab and rituximab therapy was 33.3% and reached 66.7–80% with the addition of lenalidomide. A remarkable ORR was seen in ten MCL patients after durvalumab and ibrutinib combination therapy. The combination treatment of durvalumab, rituximab and bendamustine led to an ORR of 88.9% in FL patients and 30% in DLBCL patients. On the other hand, none of FL (*n* = 5), MCL (*n* = 5) or DLBCL (*n* = 10) patients responded to durvalumab as a monotherapy. Although these early findings are encouraging, serious AEs were commonly seen in patients treated with durvalumab when administrated either alone or in combination therapy (Table 2). Another phase-2, two-arm, open-label clinical trial (NCT03003520) is ongoing to evaluate the safety, activity, and predictive biomarkers of durvalumab in combination with chemoimmunotherapy (R-CHOP) or lenalidomide plus R-CHOP, followed by Durvalumab consolidation therapy, in previously untreated subjects with DLBCL. The ORR from the evaluable patients of the durvalumab–R-CHOP arm showed that 54.10% of the patients achieved CR but 51% of the cases presented serious AEs. Finally, NCT03310619 (PLATFORM) and NCT02706405 are two studies aimed at determining the safety, tolerability, and efficacy of CAR T-cells (JCAR017 and JCAR014, respectively) in combination with durvalumab in subjects with R/R B-cell malignancies. Among the first 11 evaluable patients, investigators reported an ORR of 91%, including 64% CR. The NCT02706405 study enrolled 15 patients, in which 12 were DLBCL, 2 were high-grade B-cell lymphoma with *MYC* and *BCL2* and/or *BCL6* rearrangements, and 1 was PMBL. The ORR from the 12 evaluable patients was 50%, with 42% CR and 8% PR. Only one patient who achieved CR has relapsed. 

**Table 1:** Clinical evaluation of immune checkpoint-based therapies blockade for the treatment of B-cell lymphomas.

Targets	Drug/Regimen	Trial ID	Phase	N	Disease	Response	DOR/PFS/OS	Ref
PD-L1 CD20	Atezolizumab + Obinutuzumab + Bendamustine/ Atezolizumab + Obinutuzumab + CHOP	NCT02596971	1/2	40	FL, DLBCL	ORR = 95% CR = 75%	PFS = 74.9% OS = 86.4% (24-month)	[121]
PD-L1 CD20	Atezolizumab + Obinutuzumab + Lenalidomide	NCT02631577	1/2	20	FL	ORR = 85% CR = 80%	14.5 months	[122]
PD-L1 CD20 EZH2	Atezolizumab + Obinutuzumab/ Atezolizumab + Tazemetostat	NCT02220842	1	43	FL, DLBCL	ORR = 16% CR = 5%	PFS = 1.90 months	[123]
PD-L1 CD20 BCL2	Atezolizumab + Obinutuzumab + Venetoclax	NCT03276468	2	58	DLBCL	ORR = 23.6%	N/A	[124]
PD-L1	Mosunetuzumab/ Atezolizumab + Mosunetuzumab	NCT02500407	1	218	FL, DLBCL, t-FL, iNHL	ORR = 64.1% (iNHL)/34.7% (others) CR = 42.2% (iNHL)/18.6% (others)	92.6% (5.8 months, iNHL) 68.2% (8.8 months, aNHL)	[125]
PD-L1	Atezolizumab + CD20-TCB (RG6026)	NCT03533283	1	36	FL, DLBCL, MCL, PMBL, LPL, iNHL	ORR = 36% CR = 17%	N/A	[126]
PD-L1	Atezolizumab + KTE-C19 (Axi-cel)	NCT02926833	1/2	28	DLBCL	ORR = 75% CR = 46%	not reached	[127]
PD-L1 BTK	Durvalumab + Ibrutinib	NCT02401048	1/2	61	FL, DLBCL	ORR = 25%	PFS = 4.6 months OS = 18.1 months	[120]
PD-L1	Durvalumab + R-CHOP	NCT03003520	2	46	DLBCL	CR = 54.1%	PFS = 12 months	[128]
PD-L1	Durvalumab + JCAR014	NCT02706405	1	13	DLBCL LBCL HG-BCL	ORR = 50% CR = 42%	N/A	[129]
PD-1	Pembrolizumab	NCT01953692	1b	31	DLBCL, FL, PMBL, cHL, MM	ORR = 58.1% CR = 19.4% PR = 38.7% SD = 22.6% PD = 19.4%	DOR: not reached PFS = 11.4 months	[130]
PD-1	Pembrolizumab	NCT02650999	1/2	12	DLBCL, FL, MCL, PMBL	ORR = 27% CR = 9% PR = 18% SD = 9% PD = 64%	N/A	[131]
PD-1	Nivolumab	NCT02038933	2	121	DLBCL, B-NHL	ORR = 18% CR = 5% PR = 14% SD = 14% PD = 49%	DOR = 7.4 months	[7]
PD-1	Nivolumab	NCT02038946	2	116	FL	ORR = 4%	DOR = 114 months	[68]
PD-1	Pembrolizumab + ASCT	NCT02362997	2	31	DLBCL, PMBL, iNHL	CR = 59%	OS = 93% PFS = 58%	[102]
PD-1 CD20	Nivolumab + Rituximab	NCT03245021	1	19	FL, B-NHL	ORR = 84% CR = 47% PR = 37% PR = 5% SD = 11%	N/A	[132]
PD-1	Pembrolizumab + R-CHOP	NCT02541565	1	33	DLBCL, FL	ORR = 90% CR = 77%	PFS = 83% (2-year)	[133]
PD-1 TLR4 CD20	Pembrolizumab + G100 + Rituximab	NCT02501473 (Discontinued)	1/2	18	FL, MZL	ORR = 33.3% PR = 33.3% SD = 61.1% PD = 5.6%	N/A	N/A
PD-1	Pembrolizumab + cyclophosph. + DPX-Survivac	NCT03349450	1	17	DLBCL	2 CR, 2 PR, 2 SD	N/A	[134]
PD-1 BTK	Nivolumab + Ibrutinib	NCT02329847	1/2	144	DLBCL, FL, CLL-RT, SLL	CR = 61% PR = 14% SD = 3%	N/A	[135]
PD-1 BTK	Pembrolizumab + Acalabrutinib	NCT02362035	1/2	61	DLBCL, cHL, CLL, MM, WM	ORR = 26% CR = 7% PR = 20% SD = 30% PD = 36%	PFS = 1.9 months	[136]
PD-1 BTK PI3K	Pembrolizumab + Ibrutinib + Idelalisib	NCT02332980	2	29	FL, CLL, CLL-RT, MZL, RT, WM, SLL	ORR = 17% CR = 3% PR = 7%	N/A	[104]
PD-1 CDK	Pembrolizumab + Dinaciclib	NCT02684617	1	128	DLBCL, FL, CLL, MM	ORR = 18% 3 CR, 4 PR	DOR = 4.9 months PFS = 2.1 months	[137]
PD-1 HDAC	Pembrolizumab + Vorinostat	NCT03150329	1	30	DLBCL, PMBL, FL, cHL	ORR = 30% CR = 30%	DOR = 6 months PFS = 59%	[138]
PD-1 HDAC	Pembrolizumab + Entinostat	NCT03179930	2	22	FL, cHL	ORR = 92%	N/A	[139]
PD-1	Nivolumab + Lenalidomide	NCT03015896	1/2	10	DLBCL, FL, MCL, MZL, WM, cHL	1 CR, 2 PR, 3 PD	N/A	[138]
PD-1 CD30	Nivolumab + Brentuximab vedotin	NCT02581631	1/2	30	DLBCL, PMBL, PTCL, CTCL, MF, SS	ORR = 73% CR = 37% SD = 10% PD = 10%	DOR = not reached PFS = 63.5% (6 months)	[106]
PD-1 CD19 CD22	Pembrolizumab + AUTO3	NCT03287817	1/2	24	DLBCL, t-FL, PMBL	ORR = 75% CR = 63%	N/A	[140]
PD-1 CD19	Pembrolizumab + Tisagenlecleucel	NCT03630159	1	8	DLBCL	1 PR 2 PD	N/A	[141]
PD-1 CTLA-4	Nivolumab + Ipilimumab	NCT01592370	1/2	169	cHL, B-NHL, T-NHL, MM	ORR = 20% PR = 20% SD = 7%	DOR = not reached PFS = not reached OS = not reached	[142]
CTLA-4	Ipilimumab	NCT00089076	1/2	18	DLBCL, FL, MCL	1 CR, 1 PR	N/A	[143]
CTLA-4 CD20	Ipilimumab + Rituximab	NCT01729806	1	33	DLBCL, FL, MCL	ORR = 24%	PFS = 2.6 months, FL = 5.6 months	[144]
CTLA-4 PD-1	Ipilimumab + Nivolumab	NCT01822509	1	28	B-NHL	ORR = 32%	PFS = 1 year	[145]
CTLA-4	Ipilimumab + Lenalidomide	NCT01919619	2	11	DLBCL, FL, MCL	ORR = 73%	4.6–12 months	[146]
CD47 CD20	Hu5F9-G4 + Rituximab	NCT02953509	1b/2	115	DLBCL, iNHL	ORR = 36% (DLBCL)/61% (iNHL) CR = 15% (DLBCL)/24% (iNHL) SD = 12% (DLBCL)/24% (iNHL)	N/A	[147]
CD47 CD20	TTI-621 + Rituximab	NCT02663518	1	32	DLBCL	ORR = 29% CR = 14% (monotherapy) ORR = 24% CR= 4% (combination)	N/A	[148]
CD47 CD38	TTI-622 + Daratumumab	NCT03530683	1	19	DLBCL, MCL, FL	CR = 10% PR = 10%	N/A	[149]
CD47 CD20	ALX148 + Rituximab	NCT03013218	1	33	DLCBL, MCL, FL, MZL	ORR = 41%/62.5% CR = 9%/11%	N/A	[150,151]
CD40	Dacetuzumab	NCT00103779	1	50	DLBCL, MCL, FL, MZL	CR = 2% PR = 10% SD = 26%	N/A	[152]
CD40	Dacetuzumab	NCT00435916	2	46	Relapsed FL, DLBCL, MZL	ORR = 9% CR = 4% PR = 4% 28% SD	N/A	[153]
CD40	Lucatumumab	NCT00670592	1/2	111 (74)	FL, MZL, MCL, DLBCL	33% OR 5% CR 29% PR 52% SD	N/A	[154]
CD40 CD20	Dacetuzumab + Rituximab + chemotherapy	NCT00655837	1	30	DLBCL	ORR = 47% CR = 20% PR = 27%	PFS = 25 weeks	[155]
CD40 CD20	Dacetuzumab Rituximab + chemotherapy	NCT00529503	2	154 (101)	DLBCL, FL	67% OR 18% SD 33% CR 33% PR	not reached	[156]
CD27	Varlilumab	NCT01460134	1	90 (18)	MCL, MZL, DLBCL, CLL, cHL, TCL	SD = 16%	DOR = 6% (14-month)	[157]
CD80 CD20	Galiximab + Rituximab	NCT00363636	3	337	FL	51%	12 months	[158]
CD80	Galiximab	NCT00575068	1/2	38	FL	ORR = 63%	PFS = 11.7 months	[159]
CD80 CD20	Galiximab + Rituximab	NCT00048555	1/2	73	FL	ORR = 62%	11.7 months	[160]
CD80 CD20	Galiximab + Rituximab	NCT00117975	2	61	FL	72.1%	2.9 years	[160]
4-1BB	Urelumab	NCT01471210	1	60	DLBCL, FL, B-NHL	ORR = 6% (DLBCL)/12% (FL)/17% (others)	PFS = 8.1 weeks (DLBCL)/8.9 weeks (FL)/13.4 weeks (others)	[161]
4-1BB CD20	Urelumab + Rituximab	NCT01775631	1	46	DLBCL, FL	ORR = 10% (DLBCL)/35% (FL)	PFS = 9 weeks (DLBCL)/40.4 weeks (FL)	[161]
4-1BB CD20	Utomilumab + Rituximab	NCT01307267	1	67	FL, MCL, DLBCL	ORR = 21	PFS = 4.6 months	[162]
CD70	SGN-CD70A	NCT02216890 Terminated	1	38	DLBCL, FL, MCL	PR = 15% SD = 30%	PFS = 1.9 months	[163]

Abbreviations: FL, follicular lymphoma; t-FL, transformed follicular lymphoma; DLBCL, diffuse large B-cell lymphoma; MCL, mantle cell lymphoma; MZL, marginal zone lymphoma; B-NHL, B-cell non-Hodgkin lymphoma; iNHL, indolent B-cell non-Hodgkin lymphoma; aNHL, aggressive B-cell non-Hodgkin lymphoma; LBCL, large B-cell lymphoma; HG-BCL, high-grade B-cell lymphoma; cHL, classical Hodgkin lymphoma; CLL-RT, chronic lymphocytic leukaemia Richter transformation; PMBL, primary mediastinal B-cell lymphoma; PTCL, peripheral T-cell lymphoma; CTCL, cutaneous T-cell lymphoma; MF, myelofibrosis; SS, Sezary syndrome; MM, multiple myeloma; ORR, overall response rate; CR, complete response; PR, partial response; SD, stable disease; PD, progressive disease; DOR, duration of response; PFS, progression-free survival; OS, overall survival; NA, not available.

### 2.2. CTLA-4 Signalling and Inhibition

Cytotoxic T-lymphocyte antigen 4 (CTLA-4 or CD152) is expressed by both CD4+ and CD8+ T-cells and mediates T-cell activation together with CD28 as both receptors are homologous and share a pair of ligands, CD80 and CD86, found on the surface of APCs [164]. The interaction between CTLA-4 and both ligands is of higher affinity and avidity than CD28 and plays opposite roles. While CD28 mediates T-cell costimulation in conjunction with TCR signals, CTLA-4 and its interaction with its ligands drive the inhibition of T-cell responses, although the precise mechanisms are not fully understood [165]. Two CTLA-4 blocking antibodies have been developed: tremelimumab, the first full human CTLA-4 antibody [166], and ipilimumab, an anti-CTLA-4 IgG2 monoclonal antibody and IL-2 stimulant [167]. Both agents are able to recognise human CTLA-4 and to block its interaction with CD80 or CD86 [168], potentiating an antitumor T-cell response [169]. Though ipilimumab binds to the same epitope, with a similar affinity as tremelimumab, the higher dissociation rate of ipilimumab may indicate a dynamic binding to CTLA-4, which may provide it with an improved pharmacokinetic profile [170].

While only one ongoing trial is evaluating the efficacy of tremelimumab as a single agent and in combination with durvalumab and the JAK/STAT inhibitor AZD9150 in R/R DLBCL patients, several trials have evaluated the efficacy of ipilimumab in lymphoma patients in combination with other existing therapies (mostly rituximab and nivolumab). The first phase-1/2 trial evaluating ipilimumab in relapsed settings was launched in 2004 with 18 lymphoma patients (NCT00089076). Although only two patients could be evaluated, both had clinical responses: 1 DLBCL patient had a CR of 31+ months and 1 FL patient had a PR for up to 19 months [143]. Due to the study design of phase 1, the trial was finally terminated. Subsequently, a phase-1 clinical trial was launched in 2012 to assess the effect of ipilimumab in combination with rituximab in the same cases as the previous trial (NCT01729806). The enrolment was formed by patients with FL (*n* = 13), DLBCL (*n* = 7), MCL (*n* = 2), SLL (*n* = 2) and 9 patients with an undetermined diagnosis. At 7 weeks, toxicity was evaluated and considered as manageable (Table 2). The combination of rituximab and ipilimumab resulted in more effective B-cell depletion, together with an increase in IL-2 and TNF-α levels; both phenomenons were associated with treatment response [144]. Ipilimumab was also evaluated in patients with relapsed hematologic malignancies after an allogeneic stem cell transplant (Allo-SCT) in combination with lenalidomide (NCT01919619) or nivolumab (NCT01822509). In this last combination, only a modest antitumour activity was observed, mainly in lymphoid patients. However, substantial toxicities were also observed due to graft-vs.-host disease (GVHD) [171]. In a second trial evaluating the combination of lenalidomide and ipilimumab in 13 post-Allo-SCT patients with DLBCL, FL or MCL, a 46% CR and only one GVHD were reported, although one patient died after developing a T-cell lymph proliferative disorder after treatment [146]. The combination of ipilimumab and nivolumab is currently under study in a phase-1/2 trial in patients at high risk of recurrence after Allo-SCT (NCT02681302). Out of 31 patients, 14 have DLBCL, both primary refractory (*n =* 7) and relapsed (*n =* 7). As of 2018, 65% of patients had developed immune-related AEs of grade 2 or higher, which required treatment with systemic steroids, but no GVHD (Table 2).

**Table 2:** Adverse events of immune-checkpoint-based therapy blockade for the treatment of B-cell lymphomas.

Targets	Drug/Regimen	Trial ID	Phase	N	Disease	All-Grade AEs	Grade ≥3 AEs	SAEs and Discontinuation	Ref
PD-L1 CD20	Atezolizumab + Obinutuzumab + Bendamustine/ Atezolizumab + Obinutuzumab + CHOP	NCT02596971	1/2	40	FL, DLBCL	100% Neutropenia (52%) Constipation (43%) Fatigue (40.5%)	67%	36% DAEs 1 TRD	[121]
PD-L1 CD20	Atezolizumab + Obinutuzumab + Lenalidomide	NCT02631577	1/2	20	FL	100%	73.6%	29% SAEs 23.7% DAEs	[122]
PD-L1 CD20 EZH2	Atezolizumab + Obinutuzumab/ Atezolizumab + Tazemetostat	NCT02220842	1	43	FL, DLBCL	95% Anaemia (26%) Fatigue (23%)	47%	35% SAEs 14% DAEs	[123]
PD-L1 CD20 BCL2	Atezolizumab + Obinutuzumab + Venetoclax	NCT03276468	2	58	DLBCL		84% Lymphopenia (35%) Neutropenia (33%) TP (17.5%)	10.5% DAEs	[124]
PD-L1	Mosunetuzumab/ Atezolizumab + Mosunetuzumab	NCT02500407	1	218	FL, DLBCL, tFL, iNHL	CRS (28.4%) Neurologic AE (44%)	CRS (1.4%) Neurologic AE (3.2%)	5.5% DAEs	[125]
PD-L1 CD20	Atezolizumab + CD20-TCB (RG6026)	NCT03533283	1	36	FL, DLBCL, MCL, PMBL, LPL	CRS (42%) Pyrexia (37%) Anaemia (29%)	Neutropenia (18%) Anaemia (13%) No G ≥ 3 CRS		[126]
PD-L1	Atezolizumab + KTE-C19 (Axi-cel)	NCT02926833	1/2	28	DLBCL	100%	86% Neurologic AE (29%) CRS (4%)		[127]
PD-L1 BTK	Durvalumab + Ibrutinib	NCT02401048	1/2	61	FL, DLBCL	Diarrhoea (52%) Fatigue (46%) Nausea (34%)	56% Neutropenia 13% Dyspnea (10%)	51% SAEs 32% DAEs	[120]
PD-L1	Durvalumab + JCAR014	NCT02706405	1	13	DLBCL, HG-BCL, PMBL	CRS (38%) Neurotoxicity (8%)			[129]
PD-1	Pembrolizumab	NCT01953692	1b	31	DLBCL, FL, PMBL, cHL, MM	61% Hypothyroidism (11%) Diarrhoea (11%) Nausea (11%)	11% Neutropenia Liver disease	No DAEs No TRD	[130]
PD-1	Pembrolizumab	NCT02576990	2	53	PBML	57% Neutropenia (19%) Hypothyroidism (8%)	23% Neutropenia (13%)	2% DAEs No TRD	[108]
PD-1	Nivolumab	NCT02038933	2	121	DLBCL, B-NHL	98% Nausea (17%) Fatigue (17%) Diarrhoea (12%)	62% Neutropenia (4%) TP (3%)	12% SAEs 3% DAEs No TRD	[7]
PD-1	Pembrolizumab + ASCT	NCT02362997	2	31	DLBCL, PMBL, iNHL		79% Neutropenia (26%)	19% DAEs No TRD	[102]
PD-1 CD20	Nivolumab + Rituximab	NCT03245021	1	19	FL, B-NHL	Fatigue (74%) Infection (59%) Nausea (36%)	Lipase increased (11%) Hyperglycemia (11%) Infection (11%)	No DAEs	[132]
PD-1	Pembrolizumab + R-CHOP	NCT02541565	1	33	DLBCL, FL		43% Neutropenia (23%) Infection (10%) Syncope (10%)	13% SAEs	[133]
PD-1 TLR4 CD20	Pembrolizumab + G100 + Rituximab	NCT02501473 (Discontinued)	1/2	18	FL, MZL	100%	Abdominal pain (16%) Diarrhoea (16%) Anaemia (10%)	10% SAEs 6% DAEs	N/A
PD-1 BTK	Nivolumab + Ibrutinib	NCT02329847	1/2	144	DLBCL, FL, CLL-RT, SLL	Diarrhoea (33%) Neutropenia (31%) Fatigue (26%)	82% Neutropenia (28%) Anaemia (23%)	77% SAEs 28% DAEs No TRD	[135]
PD-1 BTK	Pembrolizumab + Acalabrutinib	NCT02362035	1/2	61	DLBCL, cHL, CLL, MM, WM		Neutropenia (15%) Anaemia (11%)	41% DAEs	[136]
PD-1 CDK	Pembrolizumab + Dinaciclib	NCT02684617	1	128	DLBCL, FL, CLL, MM	63% TP (21%) Lymphopenia (16%) Anaemia (13%)	32% Lymphopenia (13%) Neutropenia (11%) TP (8%)	3% DAEs No TRD	[137]
PD-1 HDAC	Pembrolizumab + Entinostat	NCT03179930	2	22	FL, cHL		62% Neutropenia (48%) TP (19%) Anaemia (10%)	18% SAEs 15% DAE	[139]
PD-1 CD30	Nivolumab + Brentuximab Vedotin	NCT02581631	1/2	30	DLBCL, PMBL, PTCL, CTCL, MF, SS	83% Neutropenia (30%) Peripheral neuropathy (27%)	53% Neutropenia (30%) TP (10%) Peripheral neuropathy (10%)	13% SAEs 7% DAEs No TRD	[106]
PD-1 CD19 CD22	Pembrolizumab + AUTO3	NCT03287817	1/2	24	DLBCL, tFL, PMBL		Neutropenia (89%) TP (58%) Anaemia (47%)		[140]
PD-1 CD19	Pembrolizumab + Tisagenlecleucel	NCT03630159	1	8	DLBCL	100% CRS (25%) Tachycardia (25%)	50% Anaemia (25%) Pancreatitis (25%)	No DAEs	[141]
PD-1 CTLA-4	Nivolumab + Ipilimumab	NCT01592370	1/2	65	cHL, B-NHL, T-NHL, MM	Fatigue (26%) Pyrexia (23%) Diarrhoea (18%)	29%	48% SAEs 8% DAEs No TRD	[142]
CTLA-4	Ipilimumab	NCT00089076	1/2	18	DLBCL, FL, MCL	100% Diarrhoea (56%) Fatigue (56%) TP (28%)	44.4% Diarrhoea (28%) Fatigue (6%) Neutropenia (6%)		[143]
CTLA-4 CD20	Ipilimumab + Rituximab	NCT01729806	1	33	DLBCL, FL, MCL	Fatigue (33%) Anaemia (30%) Diarrhoea (15%)	Lymphopenia (18%) Diarrhea (12%) Anaemia (12%)		[144]
CTLA-4	Ipilimumab	NCT01822509	1	28	B-NHL		TP (27%) Chronic GVHD of liver (10%) Anaemia (7%)	18% DAEs	[145]
CTLA-4	Ipilimumab + Lenalidomide	NCT01919619	2	11	DLBCL, FL, MCL, others		Neutropenia (44%) GVHD (9%)		[146]
CD47 CD20	Hu5F9-G4 + Rituximab	NCT02953509	1b/2	115	DLBCL, iNHL	Infusion reaction (38%) Headache (34%) Fatigue (30%)	Anaemia (15%)	7% DAEs	[147]
CD47 CD20	TTI-621 + Rituximab	NCT02663518	1	32	DLBCL	Infusion reaction Transient TP			[148]
CD47 CD38	TTI-622 + Daratumumab	NCT03530683	1	19	DLBCL, MCL, FL	Abdominal pain (10.5%) Fatigue (10.5%) Nausea (10.5%)	Neutropenia (10.5%) No G ≥ 3 anaemia or TP		[149]
CD47 CD20	ALX148 + Rituximab	NCT03013218	1	33	DLCBL, MCL, FL, MZL	79% Rash (18%) Fatigue (9%)	Neutropenia (6%)		[150,151]
CD40	Dacetuzumab	NCT00103779	1	50	DLBCL, MCL, FL, MZL	98% Fatigue (28%) Headache (20%) Pyrexia (18%)	30%	26% SAEs No TRD	[152]
CD40	Dacetuzumab	NCT00435916	2	46	FL, DLBCL, MZL	98% Fatigue (41%) Headache (35%) Chills (33%)	46%	39% SAEs	[153]
CD40	Lucatumumab	NCT00670592	1/2	111 (74)	FL, MZL, MCL, DLBCL	100% Chills (39%) Pyrexia (34%) Fatigue (25%)	65% Lipase elevation (25%)	28% SAEs	[154]
CD40 CD20	Dacetuzumab + Rituximab + chemotherapy	NCT00655837	1	30	DLBCL	100% CRS (61%) Nausea (36%) TP (36%)	21% TP (6%) AST/ALT elevation (3%)	45% SAEs 1 TRD	[155]
CD40 CD20	Dacetuzumab Rituximab + chemotherapy	NCT00529503	2	154 (101)	DLBCL, FL		80%	44% SAEs 8% DAEs	[156]
CD27	Varlilumab	NCT01460134	1	90 (18)	MCL, MZL, DLBCL, CLL, cHL, TCL	59% Fatigue (24%) Anaemia (12%)	3% ALP elevation		[157]
CD80 CD20	Galiximab + Rituximab	NCT00363636	3	337	FL	Pyrexia (18%) Anaemia (12%)		No TRD	[158]
CD80	Galiximab	NCT00575068	1/2	38	FL	60% Fatigue (32%) Nausea (14%) Headache (11%)	3% Axillary pain (3%) Venous thrombosis (3%)	No SAEs No DAEs	[159]
CD80 CD20	Galiximab + Rituximab	NCT00048555	1/2	73	FL	96% Lymphopenia (48%) Leukopenia (36%) Fatigue (36%)	26% Lymphopenia (14%) Leukopenia (3%) Anaemia (3%)	13% SAEs 1 possible TRD	[159]
CD80 CD20	Galiximab + Rituximab	NCT00117975	2	61	FL	13% Lymphopenia Leukopenia Neutropenia	13% of events Lymphopenia		[160]
4-1BB	Urelumab	NCT01471210	1	60	DLBCL, FL, B-NHL	52% Fatigue (15%) Neutropenia (12%)	15%	3.3% DAEs	[161]
4-1BB CD20	Urelumab + Rituximab	NCT01775631	1	46	DLBCL, FL	72% Fatigue (20%) AST/ALT elevation (15/13%)	28%	2% TRD (CRS)	[161]
4-1BB PD-1	Urelumab + Nivolumab	NCT02253992	1/2	22	DLBCL	63% Fatigue (26%) ALT/AST elevation (13/9%)	ALT/AST elevation (3/3%)	7% DAEs	[172]
4-1BB CD20	Utomilumab + Rituximab	NCT01307267	1	67	FL, MCL, DLBCL	95.5% Fatigue (16%)	3% Neutropenia Diarrhoea ALT elevation	4.5% DAEs No TRD	[162]
CD70	SGN-CD70A	NCT02216890 Terminated	1	38	DLBCL, FL, MCL	100% TP 75% Anaemia 50%	90% TP 65%	55% SAEs	[163]

Abbreviations: FL, follicular lymphoma; tFL, transformed follicular lymphoma; DLBCL, diffuse large B-cell lymphoma; MCL, mantle cell lymphoma; MZL, marginal zone lymphoma; B-NHL, B-cell non-Hodgkin lymphoma; iNHL, indolent B-cell non-Hodgkin lymphoma; aNHL, aggressive B-cell non-Hodgkin lymphoma; LBCL, large B-cell lymphoma; HG-BCL, high-grade B-cell lymphoma; cHL, classical Hodgkin lymphoma; CLL-RT, chronic lymphocytic leukaemia Richter transformation; PMBL, primary mediastinal B-cell lymphoma; PTCL, peripheral T-cell lymphoma; CTCL, cutaneous T-cell lymphoma; MF, myelofibrosis; SS, Sezary syndrome; MM, multiple myeloma; AE, adverse event; SAE, serious adverse event; TP, thrombocytopenia; AST, alanine aminotransferase; ALT, alanine aminotransferase; ALP, alkaline phosphatase; CRS, cytokine release syndrome; GVHD, graft-versus-host disease; TRD, treatment-related death; DAE, discontinuation due to adverse event; NA, not available.

### 2.3. CD47 Signalling and Inhibition

#### 2.3.1. Overview of the Pathway

CD47 (cluster of differentiation 47) is an integrin-associated molecule belonging to the Ig superfamily [173] that interacts with SIRPα (signal regulatory protein-alpha), spreading the “don’t eat me” signal to macrophages, a strategy employed as an immune-mediated clearance evasion mechanism in several types of cancers [174] (Figure 1). Mechanistically, the CD47-SIRPα binding leads to tyrosine phosphorylation of SIRPα immunotyrosine inhibitory motifs and activates SHP (tyrosine phosphatases Src homology 2 (SH2)-containing protein tyrosine phosphatase)-1 and -2. The interaction of the phosphatase SH2 domains to phosphorylated SIRPα disrupts their autoinhibitory activity, triggering enzymatic activity and, ultimately, leading to the blockade of macrophage phagocytic function [175,176]. 

Although CD47 is widely expressed on the surface of a broad range of cell types, high levels of CD47 have also been observed in haematological cancers such as acute myeloid leukaemia (AML), ALL, CLL, multiple myeloma (MM), myelodysplastic syndrome (MDS), DLBCL, MCL, and MZL [174,177,178,179,180,181,182]. High levels of CD47 are considered to be an adverse prognostic indicator of survival [174].

#### 2.3.2. CD47-SIRPα Axis Inhibition 

One of the first attempts to target CD47 was carried out therapeutically in AML primary human xenograft models [174]. The CD47 antibody B6H12 induced phagocytosis and eliminated AML stem cells. Subsequently, it was demonstrated that this antibody synergised with anti-CD20 (which can also bind Fc-receptors), promoting a more potent prophagocytic signal in B-NHL xenograft models [179,180,183]. Based on these results, the humanised anti-CD47 antibody Hu5F9-G4 was developed [184]. Preclinical studies showed that this antibody could bind specifically to CD47, blocking CD47–SIRPα interaction and enabling macrophage-mediated phagocytosis in primary AML cells. This antibody was further shown to potently synergise with rituximab in B-NHL xenografts, supporting its evaluation in a phase-1 clinical trial (NCT02216409) that revealed its safety, pharmacokinetics, and pharmacodynamics [185]. Subsequently, a larger phase-1b/2 study was launched to evaluate the combination of Hu5F9-G4 with rituximab in 115 B-NHL patients (70 DLBCL and 45 iNHL (41 FL and 4 MZL); NCT02953509). In this trial, a Hu5F9-G4 and rituximab combination was well tolerated, with rapid and durable responses [147,186] (Table 1). 

Recently, the fully human anti-CD47 IgG4 antibody TJC4 (TJ011133) was shown to specifically block the CD47-SIRPα axis, enhancing phagocytosis in a set of tumour cell lines and AML primary cells. In BL and DLBCL xenograft models, TJC4 inhibited tumour growth and extended mice OS as monotherapy. When combined with rituximab, the antibody showed superior efficacy in a DLBCL model over the single agent. In addition, single-dose or repeat-dose treatment of TJC4 minimally affected red blood cells in cynomolgus monkeys, with no impact on platelets [187]. 

TTI-621 is a fully human recombinant fusion protein based on the structure of SIRPα linked to the Fc region of human IgG1; it was conceived and designed as a decoy receptor. First, in vitro data showed that TTI-621 could bind CD47 and induce a potent macrophage-mediated antibody-dependent cell phagocytosis (ADCP) and apoptosis in an extensive range of hematologic and solid tumour cells [188,189,190]. In vivo data indicated that the fusion protein was able to block CD47 and impair the tumour growth in several haematological xenograft models, including AML, BL and DLBCL. Preclinical data also suggested that TTI-621 was less likely to evoke anaemia when compared to other anti-CD47, thanks to its low erythrocyte–binding profile [188]. TTI-621 was also suggested to enhance the adaptive immune response [188,191]. A phase-1, open-label, multicentre study is currently ongoing to evaluate the activity of TTI-621 as a single agent or in combination with rituximab in R/R cohorts of haematologic malignancies (NCT02663518; Table 1) [148]. Another set of preclinical data show TTI-622, a new human SIRPα linked to human IgG4, induces ADCP in a panel of haematological and solid tumour cells, with a superior affinity for tumour cells than for platelets. In vivo DLBCL xenograft models indicated that TTI-622 treatment leads to a decrease in tumour growth and improves survival [192]. Based on these data, a phase-1 dose-escalation study was initiated (NCT03530683). As of April 2020, 19 R/R lymphoma patients have been enrolled (*n* = 10 DLBCL, *n* = 5 HL, *n* = 1 FL, *n* = 1 MCL, *n* = 2 peripheral T-cell lymphoma (PTCL)) and objective response has been reported in 2 DLBCL patients (1 PR and 1 CR) [149].

A newly engineered high-affinity SIRPα-Fc fusion protein, ALX148, was able to trigger both innate and adaptive antitumor immune responses, characterised by an enhancement on phagocytosis. In an MCL xenograft mice model, although ALX148 was able to inhibit tumour growth, superior activity was observed by combining this agent with obinutuzumab. Similarly, in a BL xenograft mice model, the combination of ALX148 with rituximab enhanced tumour growth inhibition (TGI) and improved mice survival when compared to the control group [193]. Currently, ALX148 is being investigated in a phase-1 dose-escalation/expansion in patients with R/R B-NHL patients (NCT03013218). Preliminary data showed that ALX148 is well tolerated, with ORR ranging from 41% (9% CR) to 62.5% (11% CR) [150,151].

Assuming that CD47 is upregulated in both tumour cells and erythrocytes and platelets, it is understandable that targeting CD47 leads to side effects, including anaemia. To get through such unwanted effects, a fully human bispecific antibody, TG-1801 (NI-1701), comprising a high-affinity CD19-targeting arm combined with CD47-blocking arms, with a range of affinities on a human IgG1 Fc backbone, was developed [194]. In vitro TG-1801 specifically and strongly binds to human B-cells, avoiding hemagglutination. The specific blockade of the CD47-SIRPα axis on CD19-expressing cells mediates effective killing of primary and immortalised B-NHL cells via ADCP and antibody-dependent cell cytotoxicity (ADCC) [195,196]. Moreover, the bispecific antibody prevented the recruitment of CD19 to the BCR signalling complex, and the coligation of CD19 and CD47 by TG-1801 limited CD19 mobility at the B-cell surface by the cytoskeleton-anchored glycoprotein CD47, inhibiting B-cell proliferation and BCR-mediated gene expression [197]. While TG-1801 has demonstrated to be superior to rituximab in killing B-cells from primary leukaemia and lymphoma cells [196], its combination with the novel glycoengineered anti-CD20 mAb ublituximab or U2-regimen-associating ublituximab, with the dual PI3Kσ/CK1ε inhibitor umbralisib, allowed a synergistic effect in both ADCC and ADCP [198,199]. In vivo xenograft BL and B-ALL models showed that TG-1801 reduced tumour growth and also increased survival time [196]. Complementarily in DLBCL patient-derived xenografts (PDX), the antibody reduced tumour burden, with significantly higher efficacy than ibrutinib [200]. Lastly, the TG-1801–U2 combination has shown synergistic activity in-vivo in a BL xenograft model, associated with infiltration of effector cells (NK and macrophages) [198,199]. Based on the preclinical data, TG-1801 is currently in a phase-1 trial (NCT03804996) for histologically confirmed B-cell lymphoma, relapsed or refractory to prior standard therapy.

### 2.4. CD40 Signalling and Inhibition

CD40, a member of the TNF receptor family expressed by APCs (DCs, macrophages, NK cells, and mature B-cells), interacts with its ligand CD40L (CD154), which is expressed by activated T-cells, stimulating cytokine secretion by B-cells and allowing T-cell activation [201,202]. CD40 activation promotes the conversion of DCs to APCs, the phagocytic ability of macrophages, and proliferation and antigen presentation on B-cells [203]. CD40 is expressed in a wide range of B-NHL, CLL and MM [204]. 

CDX-1140 is a novel agonist antibody against CD40, binding outside of the CD40L ligation site. Preclinical data showed enhanced DC and B-cell activation by CDX-1140, which synergises with recombinant CD40L to enhance agonist activity [205]. While xenograft models using CD40+ lymphoma cell lines have shown antitumour activity by CDX-1140, with attenuated tumour growth and increased survival, safety studies in cynomolgus macaques support the use of the antibody in humans [203,205,206]. A phase-1 trial (NCT03329950) is currently recruiting and will evaluate the safety and efficacy of CDX-1140 alone or in combination with the soluble recombinant Flt3 ligand CDX-301, pembrozilumab or chemotherapy (gemcitabine and nab-paclitaxel) [207]. 

Selicrelumab is an agonist antibody that activates both memory and naïve B-cells and triggers T-cell activation [208]. Preclinical studies, both in vivo and in vitro, resulted in antitumour activity via an immune activation; a synergy was observed in vivo when combined with chemotherapy agents or in a triple-combination with PD-L1 inhibition and the FAP-IL2v immunocytokine [209]. A phase-1 clinical trial is ongoing (NCT03892525), with an estimated enrolment of 44 patients, to assess selicrelumab’s safety profile in combination with atezolizumab in patients with R/R lymphoma.

Ad-ISF35 is a replication-defective adenovirus vector that encodes for the chimeric protein CD154. Its induction results in an antitumour response associated with macrophage infiltration and an increased proinflammatory cytokine release that will lead to a break in tumour immune tolerance and tumour regression [210,211]. Both in vitro and in vivo assays have shown safer administration and significant antitumoral activity as a single agent. In parallel, combinations of this agent with anti-PD1 or a triple-combination with an anti-PD1 and an anti-CTLA-4 have shown synergistic effects in melanoma [212].

Dacetuzumab, also known as SGN-40, provides inhibitory proliferation and apoptosis signals in high-grade B-NHL. Its signalling contributed to cell death by the degradation of BCL-6 and an increased expression of proapoptotic proteins [213,214,215]. A phase-1 clinical trial (NCT00103779) [152] was completed with 50 patients of refractory or recurrent B-cell lymphomas, and a phase-2 clinical trial (NCT00435916) [153] was completed with 46 relapsed DLBCL patients; however, due to its modest effect as a single agent, clinical trials were continued as a combination with other immune checkpoint inhibitors. A phase-1 clinical trial (NCT00655837) was completed, with 30 patients receiving dacetuzumab in combination with rituximab and chemotherapy (gemcitabine) [155]. A phase-2 clinical trial (NCT00529503) was completed with 154 DLBCL and FL patients with improved OR when combined with rituximab and chemotherapy (etoposide, carboplatin, and ifosfamide) [156].

SEA-CD40 is an agonist antibody with improved properties in vitro and in vivo when compared to dacetuzumab, as it induces more robust cytokine production and results in the activation of CD4+ and CD8+ T-cells [216,217]. A phase-1 clinical trial (NCT02376699) with an estimated enrolment of 135 patients is currently open to assess SEA-CD40’s safety profile as a single agent [218].

Lucatumumab, also known as CHIR-12.12, is an antagonist antibody that blocks CD40/CD40L interaction, thereby blocking a survival signal in B-cell lymphomas [219]. In xenograft models, the antibody reduced tumour growth and increased CD40 expression on tumour tissue [220,221]. Lucatumumab was tested in a phase-1/2 clinical trial (NCT00670592) with 74 NHL patients; nevertheless, it was discontinued in 2013 due to minimal clinical activity [154].

### 2.5. CD27 Signalling and Inhibition

CD27 is a transmembrane homodimeric phosphoglycoprotein and a member of the TNF superfamily; its ligand is CD70. It is constitutively expressed by most CD4+ and CD8+ T-cells, memory B-cells, and a portion of NK cells [222,223]. The CD27-CD70 activation on T-cells causes the activation, proliferation, survival, and maturation of the effector and memory capacity of those cells as in-vivo stimulation of CD27 with its ligand promotes strong cytotoxic T-cells responses. Naïve T-cells express CD27, and TCR signalling further upregulates its expression, suggesting a role during T-cell priming. Its stimulation on the B-cell subpopulation activates and promotes the generation of plasma cells, its proliferation, and the production of immunoglobulin [222,223,224]. Finally, it is also expressed in NK cells, where its activation induces cytolytic activity. Its expression is also detected in T-cell populations of different cancer subtypes, including B-cell malignancies, suggesting potential therapeutic targeting of CD27 immunomodulation [225].

Varlilumab (CDX-1127), is a monoclonal antibody that acts as an agonist of CD27-CD70 interaction. This anti-CD27 mAb provided costimulatory signals to human T-cells in a TCR-dependent manner and enhanced the number and activity of TILs [226,227]. Both in vitro assays and in vivo models have shown direct antitumor activity against CD27-positive lymphomas [228]. In-vivo assays, in combination with other immune-checkpoint-blocking antibodies such as anti-PD-L1 or anti-CD20 Abs, have demonstrated a synergistic antitumour activity [228,229]. A phase-1 clinical trial (NCT01460134) was completed with 25 DLBCL and FL patients to assess the safety and pharmacokinetic profiles of varlilumab [157]. Doses up to 10 mg/kg weekly were well tolerated, and the results obtained in this clinical trial support the hypothesis that combination therapy can enhance and improve the overall outcome. Nowadays, two clinical trials are active: a phase-1/2 (NCT03307746) study and a phase-2 (NCT03038672) study, with an estimated enrolment of 40 and 106 patients, aimed at evaluating varlilumab–rituximab and varlilumab–nivolumab combinations in R/R B-cell lymphoma patients, respectively [230,231].

### 2.6. CD80 Signalling and Inhibition

Cluster of differentiation 80 (CD80, B7-1) is a type I membrane protein member of the Ig superfamily that is expressed by various immune cells, from monocytes to APCs [232]. It binds to CD28 on the T-cell surface to activate the autoregulation of several functions, including CTLA-4 signalling (Figure 1). The interaction between this protein and the CD28 antigen is a costimulatory signal for the activation and proliferation of T-cells, inducing cytokine production [233]. Due to its intricate role in immune regulation, targeting CD80 for diverse B-cell lymphomas and autoimmune diseases has been attractive to both researchers and clinicians [234].

To date, only one antibody targeting CD80 has been developed; it is being evaluated in several clinical trials in B-NHL patients, specifically in FL. Galiximab (IDEC-114) is an IgG1 lambda mAb, with a high affinity to CD80. Galiximab effectively blocks CD80–CD28 interactions on T-lymphocytes but has no significant effect on CD80–CTLA-4 interactions [235]. This interaction usually leads to downregulation of T-cell activity, and it should, therefore, remain intact during galiximab therapy. Galiximab acts primarily via cross-linking of CD80 molecules and induction of ADCC, but it also inhibits cellular proliferation and upregulates apoptotic proteins [159]. In 2002, the first phase-1/2 clinical trial was launched, with the enrolment of 38 R/R FL patients (NCT00575068) [159]. In the same year, the combination of galiximab with rituximab was also evaluated in 73 patients with progressive FL that had failed at least one prior standard therapy, excluding rituximab (NCT00048555). This combination has also been evaluated as a first therapy for stages 3 and 4 or bulky FL in a 2005 clinical trial with 61 patients enrolled (NCT00117975) [160]. In 2006, a randomised phase 3 trial was initiated to evaluate if the galiximab–rituximab combination extended PFS compared to rituximab + placebo in 337 patients with grade 1–3a FL that had progressed or relapsed after at least one prior treatment (NCT00363636). One hundred seventy-five patients were given the combination and the remaining 162 were given rituximab + placebo, with 3% more incidence of side effects in the combination group [158].

### 2.7. 4-1BB Signalling and Inhibition

4-1BB (CD137, TNFRSF9) is another surface glycoprotein member of the TNF receptor superfamily that is expressed in a variety of immune cells, including T-lymphocytes and NK cells. A-1BB ligation by its natural ligand 4-1BBL (CD137L), expressed by DCs, macrophages and B-cells, among others, induces the activation of NF-kB and MAPK pathways [236], increasing survival, proliferation and effector function [236,237]. 4-1BB is considered a promising target for immunotherapy in B-NHL patients since microarray analyses have shown the overexpression of 4-1BB in DLBCL and FL biopsies [238]. Accordingly, treatment with agonistic anti-4-1BB antibodies in a mouse model of B-cell lymphoma eliminated the tumour in 60% of the animals, which became immune to a rechallenge after 100 days [238]. Stimulation of NK cell proliferation and function [239] and inhibition of Treg cell suppressive activity [240] could be contributing to the antitumoral effect of anti-4-1BB therapy as well; however, the role of 4-1BB signalling in these cell types is still controversial [241,242].

Urelumab (BMS-662513), the first anti-4-1BB agent to enter clinical trials, is an agonistic antibody that has shown costimulatory activities both in vitro and in primates [243,244]. In a phase-1 clinical trial (NCT01471210) with R/R B-NHL patients dosed with urelumab as a single agent, ORRs were modest in both DLBCL and FL patients (Table 1). Furthermore, half of the responses occurred in patients treated with urelumab 0.3 mg/kg, above the later-determined maximum tolerated dose (MTD) of 0.1 mg/kg, and toxicity was prominent (Table 2) [161].

The combination of urelumab plus rituximab was evaluated in a phase-1 clinical trial (NCT01775631) in relapsed B-NHL patients. The toxicity profile was similar to that of monotherapy, and ORRs were similar or lower than those previously reported for rituximab monotherapy (37% in DLBCL and 36–48% in FL), indicating no synergistic effect of the two drugs [161]. On the other hand, the combination of urelumab with nivolumab was well tolerated in a phase-1/2 clinical trial (NCT02253992) for refractory DLBCL patients. Again, no significant clinical benefit was found, as none of the patients achieved a response [172] despite the promising additive effect observed in animal models of solid cancers [245]. After these overall discouraging results in the clinical setting, there are currently no trials evaluating urelumab in B-NHL patients.

Utomilumab (PF-05082566) is an anti-4-1BB antibody with promising costimulatory activity in vitro and in vivo and antitumor efficacy in several solid cancer models [237,244,246]. Utomilumab monotherapy displayed manageable toxicity in a phase-1 clinical trial (NCT01307267) with 55 patients, including 2 relapsed B-NHL; however, these were not included in the efficacy analyses [247]. In the same trial, utomilumab in combination with rituximab achieved an ORR of 21% (*n* = 67 B-NHL) and presented an improved safety profile [162] that is likely due to the ability of utomilumab to block ligand binding, in contrast with urelumab [244]. Several clinical trials (NCT02951156, NCT03440567, NCT03704298) are currently evaluating this antibody in combination with other immunotherapeutic agents like avelumab, ibrutinib, CD19-CAR T-cells, or chemotherapeutic agents, but no results are available at the moment.

Two novel anti-4-1BB antibodies are being evaluated in clinical trials that include refractory B-NHL patients. The ligand-blocking agonistic antibody ADG106 has shown promising results in animal models of several cancers [248,249] and is being tested as a monotherapy in two clinical trials (NCT03707093; NCT03802955). The 4-1BB x PD-L1 bispecific antibody MCLA-145 has been developed with the specific aim of activating 4-1BB signalling in the tumour, where PD-L1 is expressed, as well as blocking immune-inhibitory signalling from the PD-1/PD-L1 axis. Antitumor efficacy has been reported in mouse models of several solid cancers [250,251], and, consequently, a phase-1 clinical trial (NCT03922204) is testing MCLA-145 as a single agent.

### 2.8. CD70 Signalling and Inhibition

CD70 is another transmembrane glycoprotein of the TNF superfamily that acts as a ligand for CD27. CD70 is transiently found on T-cells, B-cells, DCs, and also NK cells [222,223,252]. CD70 is controlled and induced by antigen receptor stimulation and its expression is under cytokine regulation; its expression is enhanced due to proinflammatory cytokines, such as IL-1a or IL12, or decreased due to anti-inflammatory cytokines like IL-4 or IL-10 [253]. The protein is also expressed in highly activated lymphocytes, and its expression was confirmed across different subtypes of T- and B-cell lymphomas but found absent in their normal counterparts [254,255]. 

SGN-CD70A is a potent antibody–drug conjugate (ADC) that consists of three functional subunits composed of an anti-CD70 antibody, a protease-cleavable linker, and a DNA-crosslinking pyrrolobenzodiazepine (PBD) dimer drug. Upon binding with its target, CD70, the complex is internalised and traffics to the lysosomes, where the drug is released and will initiate cellular events when it crosslinks DNA. The drug works by activating the DNA damage pathways, in both in-vitro and in-vivo studies, causing a G2 cell cycle arrest and high levels of DNA damage in treated cells [256]. Preclinical in-vitro assays have demonstrated that the formation of double-strand breaks (DSB) is an early event that will be followed by an inhibition of proliferation and induction of apoptosis in NHL cell lines [254,257]. SGN-70A inhibited cell growth and induced higher caspase activity in CD70-positive cell lines of cutaneous T-cell lymphoma (CTCL) and patient-derived T-cell lymphoma primary cells. A phase-1 clinical trial (NCT02216890) with 38 patients of R/R MCL and DLBCL was terminated to assess the safety profile of SGN-CD70A [163]. The treatment showed antitumor activity, but no further clinical trials were conducted due to the frequency and severity of the AEs (Table 2). 

### 2.9. LAG-3 Signalling and Inhibition

LAG-3 (lymphocyte activation gene-3), a CD4 homolog, is a member of the Ig superfamily expressed by TILs, activated CD4+ and CD8+ T-cells, regulatory T-cells, and NK, DC and B-cells [258,259,260]. LAG-3 has a high affinity for MHC class II molecules and exerts an inhibitory role on T-cell-mediated immune responses [261] and CD4+ and CD8+ memory T-cell activation [262]. Previous data has shown that LAG-3 is coexpressed with PD-1 in the development of T-cell exhaustion in viral infections [263]. In FL patients, LAG-3 expression was observed within PD-1^+^ functionally exhausted T-cells. Interestingly, the dual treatment with anti-PD-1 and anti-LAG-3 antibodies restored the T-cell function more efficiently. In a small cohort of 28 patients with FL, LAG-3 expression by T-cells was clinically relevant and related to patient outcome [264]. Recently, LAG-3 overexpression has been shown in a cohort of 163 DLBCL patients, mostly at the surface of CD4+ Tregs and CD8+ TILs. In this cohort, a high expression of LAG-3 and PD-1 was associated with inferior PFS and OS. In addition, the authors were able to identify a population of regulatory LAG3^high^ B-cells that polarise tissue-resident macrophages to promote a tolerogenic TME, which could influence the response to therapy [265]. Recently, a very innovative approach was used to unravel TME architecture in cHL by spatial-resolution–based single-cell analysis. The authors were able to identify and characterised novel cellular subpopulations, including immunosuppressive LAG3+ T-cells. Interestingly, it was observed that of LAG3+ CD4+ T-cells did not coexpress PD-1. Mechanistically, an in-vitro HL coculture system revealed crosstalk between the cytokines and chemokines released by Hodgkin and HRS cells and LAG3+ T-cells, favouring the immunosuppressive activity in the cHL TME. Furthermore, the removal of the LAG3- population in primary samples of cH could restore T-cell activity [266]. 

IMP321 was the first LAG-3 Ig fusion protein investigated in clinical trials [267]. Relatlimab (BMS-986016), the first anti-LAG-3 antibody, is being evaluated as a single agent or in combination with nivolumab in a phase-1/2a clinical trial (NCT02061761) with 132 patients with R/R B-NHL, CLL, cHL and MM. The results of this study are expected by January 2021. In parallel, a phase-2 open-label study (NCT03365791) of the humanised IgG4 mAbs spartalizumab and ieramilimab (LAG525) was conducted in patients with solid tumours and haematological cancers (*n* = 7 DLBCL). The results were assessed as a clinical benefit rate after 24 weeks, and the combination therapy showed promising activity in DLBCL patients who reach the expansion criteria [268]. Recently, a high-affinity anti-LAG-3 IgG1κ antibody, INCAGN02385, was engineered. Preclinical data showed that the antibody, alone or in combination with an anti-PD1, enhanced T-cell responsiveness to TCR stimulation. These data supported the evaluation of INCAGN02385 in early phase-1 (NCT03538028) testing in patients with advanced or metastatic cancers, including DLBCL [269]. Lastly, fianlimab (previously known as REGN3767), a human-engineered IgG4 antibody, was able to rescue T-cell activation in vitro and synergise with cemiplimab. This combination was able to surpass the inhibitory effects of MHC II/LAG-3 and PD-L1 signalling. Indeed, in a PD–1xLAG-3 knock-in mice model, REGN3767 treatment was able to reduce tumour growth and enhance the antitumor efficacy of cemiplimab [270]. Accordingly, simultaneous PD-1 and LAG-3 blockades are currently being investigated as a phase-1 study in multiple tumour subtypes (NCT03005782). Finally, a radionuclide-conjugated antibody, ^89^Zr-REGN3767, designed for immuno-PET analysis, was useful to identify the LAG-3-expressing intratumoral T-cells in a BL xenograft model and human PBMCs [271]. Following these results, an early phase-1 study is evaluating the 89Zr-DFO-REGN3767 anti-LAG-3 antibody for PET in R/R DLBCL patients (NCT04566978). 

### 2.10. TIM-3 Signalling and Inhibition

TIM-3 (T-cell immunoglobulin and mucin-domain containing-3) is an Ig superfamily member that is preferentially expressed in fully differentiated Th1 lymphocytes [272]. TIM-3 has recently emerged as an immune checkpoint receptor in cancer due to its selective expression in tumour tissue and the key role it plays in immunosuppression [273]. In DLBCL patients, an increased level of TIM-3 was observed in both CD4+ and CD8+ T-cells, which was positively correlated with tumour stages [274]. In addition, it was described that TIM-3 is coexpressed with PD-1 in the CD3+ T-cells of patients with DLBCL, and high levels were related to tumour stage and response to conventional chemotherapy [275]. More recently, in a set of DLBCL patients, it was shown that high levels of TIM-3 in tumour cells and TILs were associated with worse OS. The authors also suggested that the TME could be directly affected by TIM-3, which leads to decreased immune surveillance and tumour clearance [276]. Preclinical data pointed out the relevance of blocking TIM-3 together with PD-1, in particular, in several cancer models [277]. These data led to the clinical development of TIM-3 antibodies, which are being tested in combination with anti-PD-1/L1 mAbs. However, so far, no clinical trial is evaluating the effect of anti-TIM-3 alone or in combination therapy in patients with B-NHL.

### 2.11. OX40 Signalling and Inhibition

OX40 (CD134, TNFRSF4) is another member of the TNFR superfamily, mostly expressed in CD4+ and CD8+ T-cells. Its natural ligand, OX40L (CD252), is expressed in DCs, B-cells and macrophages, among others, and activates the NF-kB, MAPK and BCL-2/XL-dependent antiapoptotic pathways [278,279]. OX40 signalling on T-cells promotes survival and proliferation, as well as CD4+ T-cell production of IL-2, IL-5 and IFN-γ [280,281]. OX40 signalling has been shown to reduce the ability of Treg to suppress T-cell proliferation [282], setting the basis for the antitumor activity of the anti-OX40 antibody [283]. The first anti-OX40 antibody evaluated in NHL patients, MEDI-6469, is a murine antibody with demonstrated agonistic T-cell activity in animal models [284]. The combination of MEDI6469 plus rituximab was tested in a phase-1/2 clinical trial, which included 4 DLBCL cases. No responses were detected, as two patients presented stable disease and the other two patients died during the trial (NCT02205333). After early termination of this trial in 2016, MEDI-6469 has not been further evaluated in lymphoma patients.

The human OX40-agonist PF-04518600 has been reported to promote human T-cell proliferation, to increase cytokine production, to mediate Treg cell depletion ex vivo and to inhibit tumour growth in a mouse model of lymphoma [285,286]. Currently, a phase-1 clinical trial (NCT03636503) is evaluating PF-04518600 in combination with rituximab, utomilumab and avelumab in FL patients.

BMS-986178 is another human OX40-agonist with costimulatory effects observed in ex vivo human CD4+ T-cells as it enhances their T-cell effector functions and inhibits T-cell suppression by Treg cells [287,288]. After demonstrating a favourable safety profile in mice [287], BMS-986178 is currently being evaluated in a phase-1 clinical trial (NCT03410901) in combination with the TLR9 agonist SD-101 and radiotherapy in B-NHL patients, including FL, MCL and MZL, among others.

### 2.12. TIGIT Signalling and Inhibition

TIGIT (T-cell immunoglobulin and ITIM (immunoreceptor tyrosine-based inhibitory motif) domain) is an inhibitory receptor that is expressed in CD4+ and CD8+ T-cells, Tregs, and NK cells, among others. Its main ligand, CD155 (poliovirus receptor, PVR), is expressed on DCs, B-cells and macrophages [289,290]. Studies with mouse and human cells have revealed a variety of immunomodulatory functions of the TIGIT/CD155 axis, including polarization towards tolerogenic phenotypes of DCs and M2 macrophages, inhibition of NK cell functions, inhibition of proliferation and cytokine production by T-cells, and stimulation of Treg cells. Moreover, TIGIT blocks the costimulatory activity of DNAM1 (DNAX accessory molecule-1, CD226), which binds to CD155 with lower affinity than TIGIT [290].

Tumoral samples from B-NHL patients, including FL, DLBCL, MCL, MZL and CLL, present overexpression of TIGIT in CD4+ and CD8+ T-cells, which produce lower levels of proinflammatory cytokines as well as expression of the ligand CD155 in the TME [291]. In FL patients, high numbers of TIGIT+ T-cells have been associated with lower EFS and OS [292].

Two monoclonal antibodies targeting TIGIT are currently being evaluated in clinical trials with NHL patients. SEA-TGT (SGN-TGT) is a human anti-TIGIT antibody that blocks ligand binding and allows DNAM1 costimulatory signalling. This led to Treg depletion and CD8+ T-cell activation in in-vitro and in-vivo models, in which the drug induced a long-term antitumor response and made animals immune to a tumour rechallenge [293]. A phase-1 clinical trial (NCT04254107) recently tested SEA-TGT as a monotherapy in patients with cHL, DLBCL and PTCL, among others. The other anti-TIGIT antibody, tiragolumab (MTIG7192A, RG6058), is also being tested in a phase-1 clinical trial (NCT04045028), alone or in combination with rituximab, in B-NHL patients.

Following the observation that TIGIT and PD-1 are coexpressed in the intratumoral T-cells of NHL patients [291] and the favourable results of the combination of anti-TIGIT and anti-PD-1/PD-L1 in preclinical models and some clinical trials for solid cancers [289,294], the combination will, most likely, be evaluated in NHL patients in future clinical trials.

## 3. Mechanisms Underlying B-Cell Lymphoma Refractoriness to Immune Checkpoint Blockade

Resistance to immune checkpoint blockade therapy in human cancers was extensively reviewed by Bellone M and Elia A [295]; however, the mechanisms underlying B-cell lymphoma refractoriness to immune checkpoint blockade are still poorly understood.

**Figure 3:**
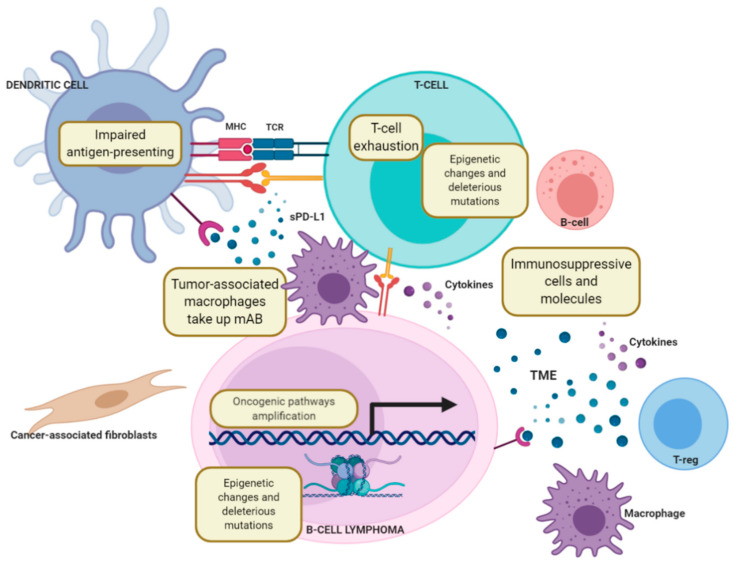
Mechanisms of resistance to immune checkpoint blockade. The deregulation of MHC class I components such as the loss of β2 microglobulin (B2M) and the loss of human leukocyte antigen (HLA) heterozygosity as well as defects in IFN signalling pathways may impair antigen recognition by antitumor CD8+ T-cells. Amplification of oncogenic signalling pathways such as PI3K/AKT/mTOR, Wnt/β-catenin, and MAPK increases the production of immunosuppressive cytokines, trigger T-cell exclusion from TME and may also result in resistance to immune checkpoint blockade. Epigenetic (histone acetylation or DNA methylation) and genetic (deleterious mutations) alterations are crucial triggers of gene expression disorders related to sustained T-cell exhaustion that could eventually cause the failure of immune checkpoint therapy. Moreover, myeloid-derived suppressor cells (MDSCs), Tregs, tumour-associated macrophages (TAMs), and cancer-associated fibroblasts (CAFs) are major immunosuppressive cell types within the TME that may contribute to resistance to immune checkpoint blockade. Immunosuppressive molecules such as TGF-β and IFN-γ, secreted by tumour cells, myeloid cells and macrophages in the TME, may also suppress the functions of effector T-cells, rendering immune checkpoint blockade ineffective.

Although major advances have been made in the last 20 years to overcome the refractoriness of B-NHL patients to standard therapies through the introduction of immune checkpoint blockades in the clinical setting, either as single agents or in combination therapies (Table 1 and Figure 2), several parameters can impair the efficacy of these new approaches. Patient-intrinsic factors such as age, sex, HLA heterozygosity or loss of β2-microglobulin (B2M), amplification of oncogenic signalling pathways, immunosuppressive cells and molecules present in the TME may impair antigen recognition and contribute to the failure of immune checkpoint blockades [296,297] (Figure 3). 

The frequent PD-L1 aberrant expression that is found among lymphoma patients results in the most responsive cancer type to anti-PD1 therapy. However, the PD-1/PD-L1 blockade can be strongly influenced by disease-specific factors, and its predictive value in clinical trials is still controversial. The inconsistent data could be attributable mainly to the variable PD-L1 resources (tumour cells, tumour microenvironment cells, peripheral blood), the differences in staining (including detecting antibodies) procedures, and positive/negative PD-L1 cut-offs. Additionally, it has been shown that PD-L1 can interact in cis with CD80 on APCs and then disrupt the binding between PD-1 and PD-L1 [298]. The functional effects of alternative binding partners also highlight the differences seen in the efficacy of PD-1/PD-L1 immunotherapy in different biological settings.

There are several mechanisms determining if a patient will respond or not to a PD-1/PD-L1 blockade. Weakly immunogenic tumours may have an insufficiently active T-cell population to respond to PD-1/PD-L1 blockade; additionally, potentially immunogenic tumours will also be resistant to PD-1/PD-L1 blockade if they develop mechanisms to suppress the activation and infiltration of T-cells after the treatment. Moreover, patients may become resistant to PD-1/PD-L1 therapy if they have insufficient reinvigoration of exhausted tumour-specific CD8+ T-cells or if they have lost target antigens or the ability to present them. Finally, some patients might initially respond to PD-1/PD-L1 blockade but become resistant if the antitumour T-cells are short-lived [299]. 

There are also epigenetic mechanisms of B-lymphoma cells driving the resistance to immune checkpoint blockades. The in vitro and in vivo data from Zheng et al. [300] showed that miR-155 overexpression enhances PD-L1 expression, reduces peripheral blood immune cells, induces CD8+ T-cell apoptosis and dysfunction via AKT/ERK dephosphorylation, and decreases the survival of DLBCL patients. It has also been shown that histone deacetylase 3 (HDAC3) is another important epigenetic regulator of PD-L1 in B-cell lymphoma as its inhibition increases PD-L1 transcription, resulting in a better clinical response to PD-L1 blockade [301]. 

The molecular mechanisms of the immune environment in regulating the efficacy of immune checkpoint blockades in B-cell lymphoma is poorly understood. It has been shown that T-cell-inflamed tumours are enriched for sensitivity to PD-1 blockade therapy [302]. Conversely, T-cell noninflamed tumours present low-infiltrating immune cells and are typically resistant to immune checkpoint blockade therapy [303]. Inflamed lymphomas are characterised by the presence of prominent T-cell infiltration [304], genetic alterations that facilitate escape from immune surveillance [78,82,305,306], and frequent mutations, resulting in hyperactivity of the NF-kB signalling pathway [78,307]. 

Hyperprogressive disease has been found in 9% of patients who received anti-PD-1/PD-L1 therapy [308,309]. The hyperprogression is associated with the elderly age of patients but not tumour burden or cancer type [308]. MDM2/MDM4 amplification and EGFR aberration are correlated with a higher risk of hyperprogression in solid cancers [309], although it was little known in lymphoma. Recent data showed that patients experiencing an hyperprogression have a higher prevalence of PD-L1^−^ disease [308]. It is predicted to be because the engagement of PD-1 with anti-PD-1 mAb inhibits but does not augment T-cell activations. Therefore, anti-PD-1 mAbs might be PD-1 agonists rather than antagonists in PD-L1^−^ status. The disease might also rapidly progress through interaction between PD-L1 and CD80 instead of PD-1 for the blocking duration by anti-PD-1 mAbs in PD-L1+ status [310]. Some polymorphisms of PD-1 could also affect the action of anti-PD-1 mAbs, and, thus, hyperprogression could be possible after PD-1 blockade [311].

## 4. Conclusions and Future Perspectives

Despite the remarkable implementation of immune checkpoint therapies in the last 5 to 6 years in determined subtypes of lymphoma, including cHL and PMBL, the applicability of these approaches in the management of R/R B-NHL has, so far, been mixed and predicting which lymphoma will respond to immune checkpoint blockade is currently not accurate. To date, pembrolizumab is the only FDA-approved agent for use with R/R B-NHL (i.e., PMBL) patients, illustrating the fact that, conceptually, PD-1 blockade in B-NHL appears to be promising and rarely related to severe immune-related AEs [7,97,103]. However, the efficacy of this approach is low, with no long-term durable responses except in PMBL, PCNSL, and PTL, which are due to alterations of chromosome 9p24.1 and the expression of PD-L1/PD-L2. To improve the efficacy of these agents, there have been great efforts made on combination immunotherapy with PD-1/PD-L1 and CTLA-4 checkpoint inhibitors. The superior outcomes of combined immunotherapy over single-agent regimens in preclinical studies, together with the approval of nivolumab plus ipilimumab, give hope to the therapeutic potential of CTLA-4 blockade and its possible combination with PD1/PD-L1 blockers.

Finally, understanding the complex interplay between malignant cells, lymphoid TME and immune-accompanying cells is mandatory in order to identify the specific lymphoma types that are vulnerable to a determined checkpoint and still requires improvement in the detection methods. To this aim, patient preselection based on accurate genomic and phenotypic examination of the TME will be necessary to identify the best target(s) of interest, either in monotherapy or in combination therapy, and facilitate the design of biomarker-driven trials.
